# Lineage dependence of the neuroblastoma surfaceome defines tumor cell state-dependent and -independent immunotherapeutic targets

**DOI:** 10.1093/neuonc/noaf012

**Published:** 2025-01-18

**Authors:** Nathan M Kendsersky, Michal Odrobina, Nathaniel W Mabe, Alvin Farrel, Liron Grossmann, Matthew Tsang, David Groff, Adam J Wolpaw, Alaa Narch, Francesca Zammarchi, Patrick H van Berkel, Chi V Dang, Yaël P Mossé, Kimberly Stegmaier, John M Maris

**Affiliations:** Division of Oncology and Center for Childhood Cancer Research, Children’s Hospital of Philadelphia, Philadelphia, Pennsylvania, USA; Division of Oncology and Center for Childhood Cancer Research, Children’s Hospital of Philadelphia, Philadelphia, Pennsylvania, USA; Department of Pediatric Oncology, Dana Farber Cancer Institute, Boston, Massachusetts, USA; Department of Biomedical and Health Informatics (DBHi), Children’s Hospital of Philadelphia, Philadelphia, Pennsylvania, USA; Division of Oncology and Center for Childhood Cancer Research, Children’s Hospital of Philadelphia, Philadelphia, Pennsylvania, USA; Division of Oncology and Center for Childhood Cancer Research, Children’s Hospital of Philadelphia, Philadelphia, Pennsylvania, USA; Division of Oncology and Center for Childhood Cancer Research, Children’s Hospital of Philadelphia, Philadelphia, Pennsylvania, USA; Division of Oncology and Center for Childhood Cancer Research, Children’s Hospital of Philadelphia, Philadelphia, Pennsylvania, USA; Perelman School of Medicine at the University of Pennsylvania, Philadelphia, Pennsylvania, USA; Division of Oncology and Center for Childhood Cancer Research, Children’s Hospital of Philadelphia, Philadelphia, Pennsylvania, USA; Division of Oncology and Center for Childhood Cancer Research, Children’s Hospital of Philadelphia, Philadelphia, Pennsylvania, USA; ADC Therapeutics (UK) Ltd, London, UK; ADC Therapeutics (UK) Ltd, London, UK; Ludwig Institute for Cancer Research, New York, New York, USA; Department of Biochemistry and Molecular Biology, Johns Hopkins Bloomberg School of Public Health, Baltimore, Maryland, USA; Department of Oncology, Johns Hopkins University School of Medicine, Baltimore, Maryland, USA; Perelman School of Medicine at the University of Pennsylvania, Philadelphia, Pennsylvania, USA; Division of Oncology and Center for Childhood Cancer Research, Children’s Hospital of Philadelphia, Philadelphia, Pennsylvania, USA; The Broad Institute of MIT and Harvard, Cambridge, Massachusetts, USA; Department of Pediatric Oncology, Dana Farber Cancer Institute, Boston, Massachusetts, USA; Perelman School of Medicine at the University of Pennsylvania, Philadelphia, Pennsylvania, USA; Division of Oncology and Center for Childhood Cancer Research, Children’s Hospital of Philadelphia, Philadelphia, Pennsylvania, USA

**Keywords:** antibody–drug conjugate, AXL, epigenetics, immunotherapy, neuroblastoma, surfaceome

## Abstract

**Background:**

Neuroblastoma is a heterogeneous disease with adrenergic (ADRN)-like cells and therapy-resistant mesenchymal (MES)-like cells driven by distinct transcription factor networks. Here, we investigate the expression of immunotherapeutic targets in each neuroblastoma subtype and propose pan-neuroblastoma and cell state-specific targetable cell surface proteins.

**Methods:**

We characterized cell lines, patient-derived xenografts, and patient samples as ADRN-dominant or MES-dominant to define subtype-specific and pan-neuroblastoma gene sets. Targets were validated with ChIP-sequencing, immunoblotting, and flow cytometry in neuroblastoma cell lines and isogenic ADRN-to-MES transition cell line models. Finally, we evaluated the activity of MES-specific agents in vivo and in vitro.

**Results:**

Most immunotherapeutic targets being developed for neuroblastoma showed significantly higher expression in the ADRN subtype with limited expression in MES-like tumor cells. In contrast, *CD276* (B7-H3) and *L1CAM* maintained expression across both ADRN and MES states. We identified several receptor tyrosine kinases (RTKs) enriched in MES-dominant samples and showed that AXL targeting with ADCT-601 was potently cytotoxic in MES-dominant cell lines and showed specific antitumor activity in a MES cell line-derived xenograft.

**Conclusions:**

Immunotherapeutic strategies for neuroblastoma must address the potential of epigenetic downregulation of antigen density as a mechanism for immune evasion. We identified several RTKs as candidate MES-specific immunotherapeutic target proteins for the elimination of therapy-resistant cells. We hypothesize that the phenomena of immune escape will be less likely when targeting pan-neuroblastoma cell surface proteins such as B7-H3 and L1CAM, and/or dual targeting strategies that consider both the ADRN and MES cell states.

**Key Points**

- Cellular plasticity influences the abundance of immunotherapeutic targets.
- Subtype-specific targets may be susceptible to epigenetically mediated downregulation.
- Immunotherapeutic targets in development, B7-H3 and L1CAM, show “pan-subtype” expression.

**Importance of the Study**

Neuroblastoma is a lethal childhood malignancy that shows cellular plasticity in response to anticancer therapies. Several plasma membrane proteins are being developed as immunotherapeutic targets in this disease. Here, we define which cell surface proteins are susceptible to epigenetically regulated downregulation during an adrenergic-to-mesenchymal cell state switch and propose immunotherapeutic strategies to anticipate and circumvent acquired immunotherapeutic resistance.

Neuroblastoma is a pediatric extracranial solid tumor that arises from deregulated development of the sympathoadrenal nervous system.^1,2^ It is characterized by clinical heterogeneity ranging from low-risk tumors that can spontaneously regress to extremely aggressive high-risk tumors that relentlessly progress despite intensive multimodal therapy. Neuroblastoma is currently the only pediatric solid tumor with a labeled immunotherapy indication for monoclonal antibodies specific to the GD2 glycolipid abundantly present on the surface of neuroblastoma cells.^3–6^ These monoclonal antibodies, however, show significant on-target, off-tumor toxicities as GD2 is expressed on pain fibers, and this glycolipid can be epigenetically downregulated as a potential mechanism of resistance.^7^ Chimeric antigen receptor (CAR)-engineered autologous T-cell therapies targeting GD2 have shown recent impressive antitumor activity in neuroblastoma^8^ and diffuse midline gliomas^9^; however, in both cases, responses are often transient.

Despite intensive chemotherapy, radiation therapy, and immunotherapy, approximately 50% of patients with high-risk neuroblastoma suffer relapse, which remains generally incurable, highlighting the need to identify new therapeutic targets. Investigators have evaluated the activity of small molecule inhibitors, radiotherapies, and immunotherapeutic agents targeting neuroblastoma-specific molecular aberrations. Lorlatinib, an inhibitor of mutationally activated anaplastic lymphoma kinase (*ALK*), has shown potent preclinical and clinical activity^10–12^ and is now being studied in a Phase 3 trial for newly diagnosed high-risk patients harboring an *ALK* mutation (NCT031226916). ALK is also the target of various immunotherapeutic strategies in preclinical development, including CARs^13,14^ and antibody–drug conjugates (ADCs).^15^ The norepinephrine transporter (NET, encoded by *SLC6A2*) is another protein of interest that is used for both imaging and therapeutic purposes.^2^ Radiolabeled ^131^I-MIBG, which is shuttled into neuroblastoma tumor cells by the NET overexpressed on the cell surface, is also being studied in the same Phase 3 trial as lorlatinib. *L1CAM* (CD171) is the gene encoding a cellular adhesion molecule overexpressed in neuroblastoma and is the target of a recent CAR T trial.^16,17^*CD276* encodes B7-H3, which is another cell surface molecule overexpressed in a variety of pediatric solid tumors. Monoclonal antibodies, ADCs, and CARs specific to B7-H3 are being explored in preclinical and clinical studies.^18–21^ GPC2 is a glypican protein anchored to the cell surface that was discovered as an oncogene in neuroblastoma and has been targeted with ADC and a CAR T-cell approaches,^22–25^ including an ongoing clinical trial (NCT05650749). It is also important to understand how heterogeneity and plasticity affect the expression of immunotherapeutic targets, so that we can prioritize the many therapeutic strategies in development, and to uncover novel targets enriched in therapy-resistant cells.

Neuroblastoma cells can exist as genetically identical but epigenetically distinct states, as originally described decades ago.^26,27^ Cells can be categorized as adrenergic (ADRN) or mesenchymal (MES) as defined by distinct epigenetically regulated core regulatory circuit (CRC) transcription factors that can transdifferentiate in cell line models.^28–33^ Single-cell studies of developing neural crest cells in mice have described these 2 developmental branches and their associated lineage-related transcription factors.^34^ The clinical relevance of this plasticity is postulated to be in therapy resistance as was recently demonstrated with downregulation of GD2 in response to GD2-directed immunotherapies.^7^ Here, we sought to define the plasticity of the neuroblastoma surfaceome in light of several emerging immunotherapeutic strategies for this disease.

## Materials and Methods

### RNA-sequencing Data

Neuroblastoma RNA-sequencing (RNA-seq) data were accessed through the Treehouse Childhood Cancer Initiative (Treehouse Tumor Compendium v11 Public PolyA, *n* = 200) and Gabriella Miller Kids First (GMKF) Pediatric Research Program (https://d3b.center/kidsfirst/, *n* = 195). We only assessed samples that were confirmed as neuroblastoma by histopathology. For differential expression analysis, Treehouse and GTEx datasets were converted to counts per million (cpm), filtered by genes with cpm >1, and normalized with the edgeR using trimmed mean of M-values (TMM) for downstream analyses.^35,36^ We also utilized our RNA-seq data for parental neuroblastoma cell lines (*N* = 38) and patient-derived xenografts (PDXs) (*N* = 30) as previously described.^37,38^ RNA-seq for ADRN-to-MES transition models (SKNBE2 and KPNYN) was obtained from the NCBI Gene Expression Omnibus (GSE180516).^7^ Single-cell RNA-seq datasets were collected from published studies^39–42^ and processed using our methods as described.^41^

### Subtype Classification

Neuroblastoma samples (Treehouse human tumors, GMKF human tumors, and neuroblastoma cell lines) were analyzed with singscore, a single-sample Gene Set Enrichment Analysis (ssGSEA) method.^43^ The gene set lists for ADRN and MES cell states were obtained from van Groningen et al.^29^

### Differential Expression

Log2TPM RNA-seq data were transformed with the *voom* function and modeled with lmfit.^44^ Contrasts were prepared between each neuroblastoma subtype. For each comparison, the linear model was evaluated with limma using empirical Bayes Statistics (eBayes) and adjustment with Benjamin–Hochberg post hoc testing.^45^ To evaluate ADRN-specific and MES-specific differentially expressed genes (DEGs), we filtered for a minimum log2 fold change > 1 and max adjusted *P* value <0.05. Pan-subtype genes were genes that were not differentially expressed. Surface proteins were annotated with the UniProt localization database (keyword “Cell Membrane”). Subtype-specific and pan-subtype genes were also intersected with the Food and Drug Administration’s Pediatric Relevant Molecular Target List for childhood cancers (https://www.fda.gov/media/120332/download).^46^

### Deconvolution

Two algorithms were used to predict cellular fractions of bulk patient RNA-seq data. quanTIseq was performed with bulk TPM data and the --tumor=TRUE flag.^47^ EPIC was performed with bulk TPM data after removing common genes in both MES-specific neuroblastoma and cancer-associated fibroblast (CAF) signatures (COL1A1, COL3A1, and SYNPO2).^48^

### ChIP-sequencing Analysis

ChIP-sequencing data for H3K4me1, H3K4me3, H3K27Ac, and H3K27me3 were obtained from the GEO database (GSE138315).^49^

### DNA Methylation Analysis

Methyl-sequencing data were obtained from the TARGET project available on the NCI Office of Cancer Genomics website (https://target-data.nci.nih.gov/Public/NBL/methylation_array/).^50^ Samples with available mRNA-sequencing data were used to determine dominant subtype (120 ADRN samples and 8 MES samples). The R package gviz was used to generate figures.

### Gene Set Enrichment Analysis

The *fgsea* and *msigdbr* packages were utilized to find gene sets associated with both ADRN and MES Treehouse and GMKF patient tumors.^51,52^

### Cell Culture

Neuroblastoma cell lines were cultured in RPMI-1640 Medium with L-glutamine with 10% FBS and 1% L-glutamine or IMDM with 20% FBS, 1% L-glut, and 1:1000 ITS+ Premix Universal Culture Supplement. When culturing cell lines with doxycycline-regulated transgenes, we used Tet-Free FBS. Transdifferentiation models (KPNYN and SKNBE2) were detailed in Mabe et al.^7^ Briefly, a PRRX1 gBlock was cloned into pINDUCER20 via Gateway cloning and further prepared into lentiviral particles for inducible overexpression in transduced cell models. These models were exposed to doxycycline (500 mg/µL) for at least 21 days to establish stably differentiated cells prior to experimentation.

For CRISPR/Cas9 knockout of *AXL*, we utilized the lentiCRISPRv2 vector with one of 2 *AXL*-targeted single guide RNAs (sgRNAs).^53^ We also cloned a nontargeting sgRNA and an off-target sgRNA. lentiCRISPRv2 constructs were independently packaged into lentiviral particles in HEK293T cells with psPAX2 and pMD2.G, according to the Lipofectamine 2000 protocol. Cell line models were transduced with lentiviral particles and polybrene (10 µg/mL) containing one of 4 lentCRISPRv2 constructs and selected with puromycin (1 µg/mL) for several weeks.

AXL overexpression was achieved with the TetR protein (pLenti-CMV-TetR-blastR) and a CMV/TO-regulated *AXL* transgene (pLenti-CMV/TO-AXL-puroR). Each construct was independently packaged into lentiviral particles, as above. Parental neuroblastoma cell lines were successively transduced with the TetR-containing lentivirus (5 µg/mL blasticidin selection), then the CMV/TO-AXL lentivirus (1 µg/mL puromycin selection). Expression of *AXL* was induced by adding 1 µg/mL of doxycycline to the culture medium.

### Sample Processing and Immunoblotting

Whole-cell lysates were prepared using RIPA Lysis Buffer System with Protease and Phosphatase Inhibitor Cocktails. Pellets were resuspended in lysis buffer and cleared by centrifugation at max speed (17 900 × *g*). Protein concentration was quantified with the Bio-Rad Protein Assay Kit II. Once quantified, 15 µg of each protein sample was prepared with Laemmli Sample Buffer and 50 mM DTT and run on a 4%–15% Criterion TGX Protein Gel (40 mA/gel). Proteins were transferred to a 0.45-µm Immobilon-P Membrane (overnight at 10 V or 1 hour at 50 V). Blocking and antibody incubations were performed in 5% Blotting-Grade Blocker in TBST. Membranes were visualized using ECL Plus or SuperSignal West Femto ECL on the Azure Biosystems Sapphire™ Biomolecular Imager.

### Flow Cytometry

Neuroblastoma cell lines were counted and distributed into FACS tubes for various staining conditions and an unstained control (0.5 M cells/condition). Staining solutions were prepared according to the manufacturer’s recommended antibody and LIVE/DEAD Fixable stain per assay. After all staining, washes, and fixation with 1% formaldehyde, samples were run on the CytoFLEX LX Flow Cytometer. Data were analyzed using FlowJo® software.

### Cytotoxicity Assays

Small molecule inhibitors were obtained from Selleck Chemicals. AXL-targeting (ADCT-601) and control (B12-PL1601) ADCs were studied in collaboration with ADC Therapeutics.^54^ Neuroblastoma cell lines were seeded in a 96-well plate 1 day prior to treatment with small molecule inhibitors (128 pM–50 µM) or ADCs (0.667 fM–66.7 nM). At the study endpoint (4 days for inhibitors and 5 days ADCs), cellular viability was determined using the CellTiter-Glo 2.0 Assay protocol. Each experiment was plated in technical triplicate, and data are representative of at least 2 independent experiments.

### In Vivo Studies

For murine efficacy studies, we engrafted CB17 severe combined immunodeficiency (SCID) mice with cell line-derived xenografts (CDXs) or PDXs and followed the standard protocol established in the Pediatric Preclinical Testing Consortium (now named Pediatric Preclinical In Vivo Testing [PIVOT]).^19^ In brief, mice with tumors of enrollment size (0.2–0.3 cm^3^) received 1 mg/kg of either ADCT-601 or B12-PL1601 via tail vein injection. Studies were performed with 6 animals per arm, and the mice were monitored for 100 days or until their tumor burden reached 2.0 cm^3^.

## Results

### Human Neuroblastoma Tumors and Cell Lines Classified as ADRN- or MES-Dominant Display DEGs Beyond Previously Established Gene Signatures

**Figure 1.**
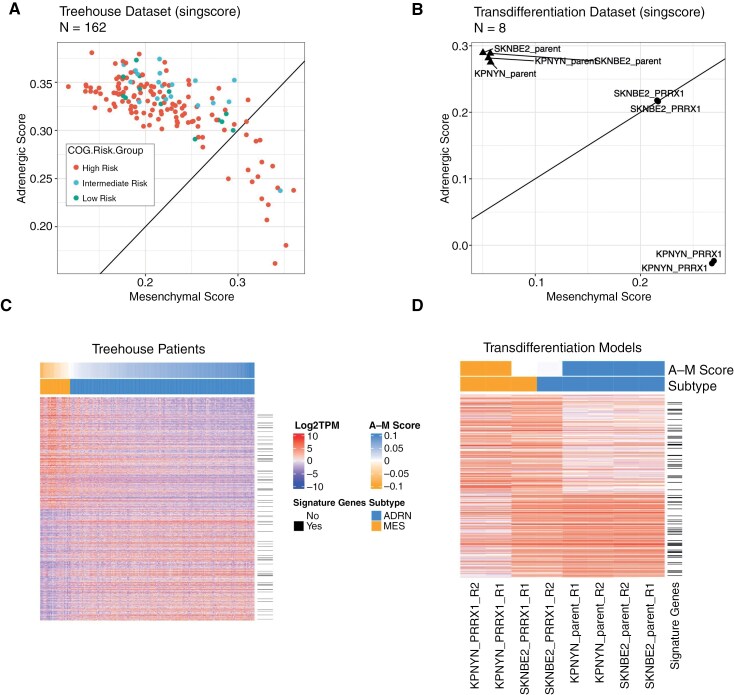
Human neuroblastoma tumors and cell lines classified as adrenergic (ADRN)- or mesenchymal (MES)-dominant display differentially expressed genes in addition to previously established gene signatures. (A, B) Single-sample Gene Set Enrichment Analysis and published ADRN/MES gene signatures were used to generate ADRN and MES scores to classify (A) Treehouse patient samples and (B) ADRN-to-MES transdifferentiation models as ADRN- or MES-dominant. Patient samples are colored by COG Risk Group. Transition models represent 2 replicates of each model (SKNBE2 and KPNYN; parent and PRRX1). (C) Heatmap displaying the top 500 MES and top 500 ADRN differentially expressed genes, as calculated by limma/voom. (D) Heatmap displaying data from transdifferentiation models, filtered for differentially expressed genes from the Treehouse patient dataset. ADRN/MES signature genes are denoted by a horizontal black line. A-M (ADRN minus MES) scores and subtypes were defined by singscore.

Prior studies defined a 485-gene MES signature and a 369-gene ADRN signature, and these have been widely used to characterize these states.^29^ However, these signatures were derived from a relatively small number of cell lines. We investigated if we could use these signatures to identify ADRN and MES tumors and cell lines in larger datasets, and then analyze those datasets to identify additional genes expressed in a state-specific manner. We assessed 2 datasets including the predominantly high-risk samples in the Treehouse Childhood Cancer Initiative (Treehouse) and slightly non-high-risk tumor-biased case series in the GMKF dataset (Supplementary Figure 1A). Using ssGSEA (singscore), we evaluated the ADRN and MES score for each tumor and characterized each sample by the dominant signature (Figure 1A, Supplementary Figure 1B). Most tumors in the Treehouse and GMKF databases were predominantly ADRN (88.4% and 92.8%, respectively), though several tumors in each dataset were MES-dominant. Within each patient dataset, risk group was not appreciably different between ADRN and MES subsets (Supplementary Figure 1C). We also determined the dominant subtype in a neuroblastoma cell line dataset^37^ and the neuroblastoma models in the Cancer Cell Line Encyclopedia (CCLE),^55^ finding that 94.7% and 83.3% were classified as ADRN, respectively (Supplementary Figure 1D and E).

We also evaluated top ADRN and MES DEGs in an ADRN-to-MES transdifferentiation (or transition) model, whereby inducible overexpression of the *PRRX1* MES transcription factor can convert ADRN cell lines to a MES-like phenotype.^7^ By singscore, both SKNBE2 and KPNYN parental cell lines assayed in this system were ADRN, with average ADRN and MES scores of 0.243 and 0.0552, respectively (Figure 1B). Upon overexpression of the *PRRX1* transgene, SKNBE2 cell lines showed a slightly decreased mean ADRN score (0.217) and significantly increased mean MES score (0.216), classifying SKNBE2-PRRX1 as a mixed phenotypic model. KPNYN cell lines overexpressing PRRX1 transitioned completely from ADRN to MES, with average ADRN and MES scores of −0.0253 and 0.269, respectively.

To further characterize neuroblastoma tumor subtypes, we performed differential gene expression between ADRN- and MES-predominant neuroblastoma Treehouse samples with limma/voom methodologies. We observed 4380 MES and 4199 ADRN DEGs using a filtering strategy of log2 fold change >1 and adjusted *P* value <0.05. The MES DEGs included 320 of 485 (83.1%) MES signature genes, and the ADRN DEGs included 153 of 369 (41.5%) ADRN signature genes. Of the top 1000 DEGs between ADRN- and MES-dominant Treehouse samples, only 75 were present in the published ADRN/MES gene signatures (Figure 1C), suggesting there are more features defining these distinct phenotypes. We also show that the same ADRN and MES DEGs in Treehouse samples were differentially expressed in the GMKF and cell line datasets (Supplementary Figure 1F). The ADRN and MES DEGs obtained from the patient dataset are also reflected in the transdifferentiation models (Figure 1D).

We next sought to validate these findings with histone ChIP-sequencing data because neuroblastoma subtypes are epigenetically mediated. We evaluated the loci of DEGs for the presence of active enhancer/promoter (H3K27ac) and repressive (H3K27me3) histone marks (Supplementary Figure 1G). We showed that enhancer marks bound to subtype-specific DEGs, where H3K27ac marks are preferentially deposited at ADRN loci in ADRN models and MES loci of the MES model. As expected, ADRN DEGs are bound by repressive marks in the MES cell line model, while the loci of MES DEGs are repressed in ADRN models. Altogether, this suggests that these patient samples and cell line models display similar transcriptional and epigenetic states, which can help us better understand the ADRN and MES phenotypes as they relate to relevant therapeutic approaches.

As the majority of DEGs were not in the published ADRN/MES gene signature,^29^ we performed GSEA to identify pathways associated with neuroblastoma patient tumors and transition cell line models (Supplementary Figure 2). We assessed the Hallmark gene sets and C2 curated gene sets (including BIOCARTA, KEGG, REACTOME) from the Molecular Signatures Database (MSigDB). In both MES-dominant patient samples and transition models, the top gene sets were related to immune cell signaling, including TNF-α, IFN-γ, and inflammatory response (Supplementary Figure 2), which validated recently published data.^56,57^ ADRN-dominant patient samples were enriched in cell cycle-related gene sets, including Hallmark pathways for E2F targets and G2/M checkpoints, as well as Reactome pathways for cell cycle checkpoints, G2/M checkpoints, M-phase, and others (Supplementary Figure 2).

We performed deconvolution with quanTIseq and EPIC to evaluate differences in immune cell subsets between MES and ADRN tumors. While some of our findings were confounded by the presence of immune cell signatures in cell lines (Supplementary Figure 3A), our analysis using EPIC showed ADRN-dominant tumors were composed of more tumor cells (7.75e-07) and MES-dominant tumors were enriched in macrophages (2.69e-03) and CAFs (*P *= 2.32e-06) (Supplementary Figure 3B). The finding of MES-enriched CAFs supports previous publications describing an association between M2 tumor-associated macrophages and CAFs, which increased the chemotherapeutic resistance of neuroblastoma tumor cells in vitro.^58^

**Figure 2.**
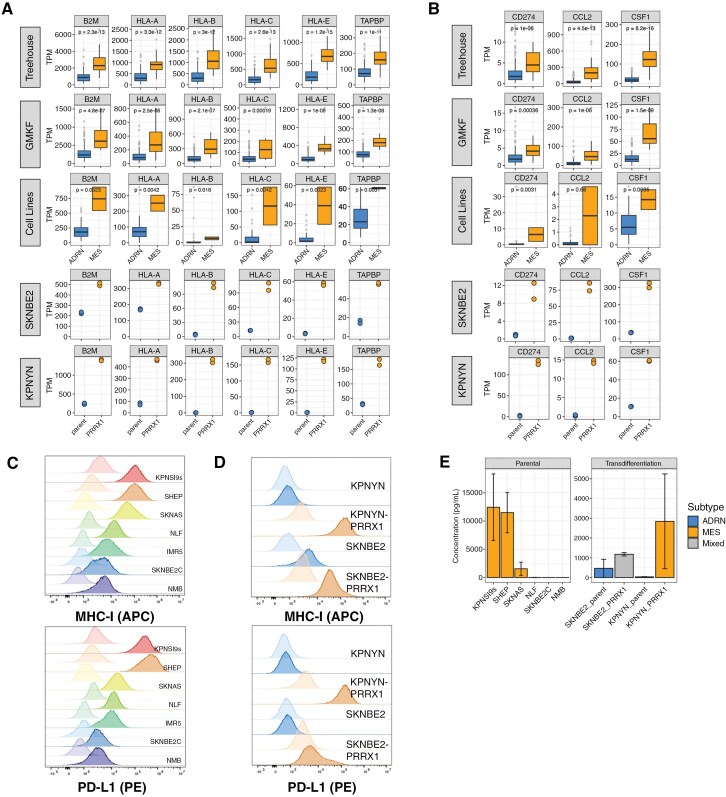
Immune signatures, including antigen presentation pathway and immunomodulatory cytokines, are enriched in mesenchymal (MES)-like neuroblastoma samples. (A, B) RNA expression of (A) antigen presentation pathway genes in Treehouse patient samples, cell lines, and SKNBE2 and KPNYN before (parent) and after (PRRX1) transdifferentiation. Statistics represent Wilcoxon test using compare_means() function in R. (C, D) Cell surface staining of MHC-I and PD-L1 in (C) parental and (D) adrenergic (ADRN)-to-MES transition models. Lighter colors represent unstained control. (E) Quantification of extracellular CCL2 in (left) parental and (right) transdifferentiation cell line models (*N* = 2, error bars show standard error of the mean).

Next, we sought to validate the MES-associated immune-related pathways in vitro. In addition to B2M and HLA-A/B/C, which were previously identified in the MES signature,^29^ we showed MES-specific expression of additional antigen processing and presentation genes (*HLA-E* and *TAPBP*), the immunomodulatory protein PD-L1 (*CD274*), and cytokines CCL2 and CSF1 (Figure 2A and B). This pattern was also observed at the epigenomic level, as *HLA-A*, *HLA-B*, *HLA-C*, *HLA-E*, *CD274*, and *CCL2* were bound by H3K27ac markers only in the SKNAS MES model (Supplementary Figure 4). Conversely, ADRN cell lines were bound by repressive histone marks at these same loci. We validated cell surface expression of MHC Class I and PD-L1 in MES-like parental cell lines and the ADRN-to-MES transition models (Figure 2C and D), as well as increased CCL2 secretion in MES parental and transition cell line models (Figure 2E).

Given that MYCN negatively regulates inflammatory response genes and immune signaling,^56,59^ we sought to understand the relationships between MYC(N) and these features in our data. In both Treehouse and GMKF datasets, MYCN and MYC are enriched in ADRN- and MES-dominant samples, respectively (Supplementary Figure 5A). Across neuroblastoma cell lines, the subtype-specific difference in MYCN and MYC are not significant (Supplementary Figure 5A). Interestingly, transdifferentiation of SKNBE2 shows the expected subtype-specific MYCN-to-MYC expression, while both MYCN and MYC are downregulated in the KPNYN model (Supplementary Figure 5B). Of note, KNPYN shows complete repression of MYCN with maintained MYC expression, while SKNBE2 retains high MYCN expression after induction of PRRX1. At the MYC locus, we show H3K27ac signal in MES and H3K27me3 in ADRN models, supporting subtype-specific expression (Supplementary Figure 5C). The MYCN locus shows both H3K27ac and H3K27me3, though the ADRN models shown are MYCN-amplified which complicates the interpretation (Supplementary Figure 5C). Together, these data suggest that pathways critical for adaptive immunity are suppressed in the ADRN cell state, likely through MYCN, and are upregulated in MES-like neuroblastoma cells.

### Most Known Cell Surface Therapeutic Targets Are Differentially Expressed Between Neuroblastoma ADRN and MES Subtypes

**Figure 3.**
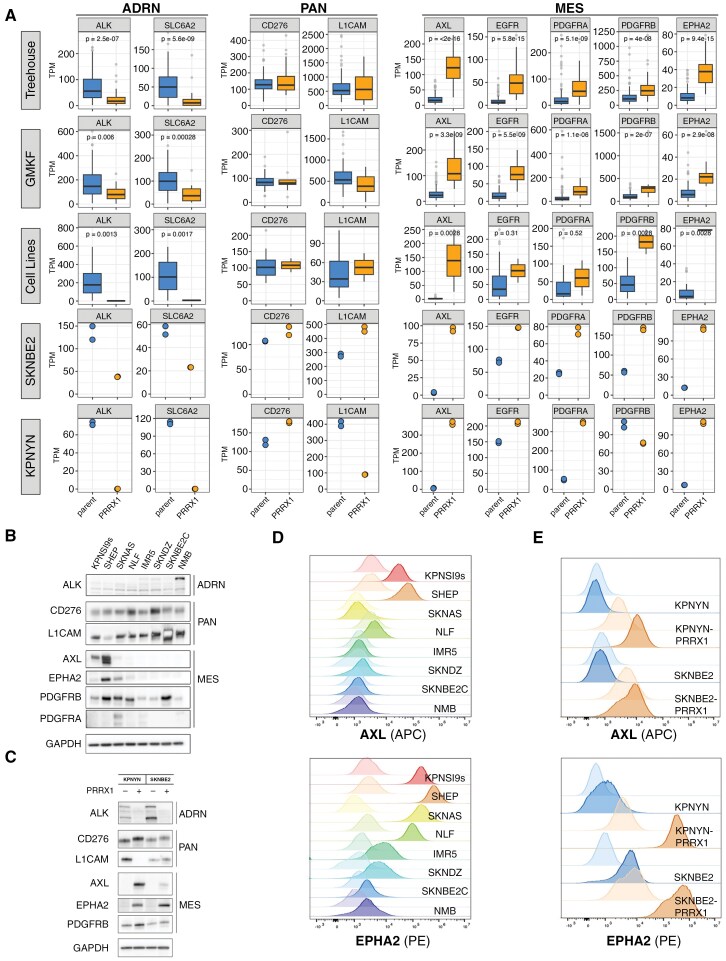
Differential expression reveals adrenergic (ADRN)-specific, pan-subtype, and mesenchymal (MES)-specific targets. (A) RNA expression of ADRN-specific, pan-subtype, and MES-specific targets in patient tumors, parental cell lines, and ADRN-to-MES transition models (SKNBE2 and KELLY). Statistics represent Wilcoxon test using compare_means() function in R. For ADRN-specific genes, outliers above the upper whisker limit (1.5 * IQR) were removed from plot visualization. (B, C) Protein expression of select targets in (B) parental and (C) ADRN-to-MES transdifferentiation cell line models. (D, E) Cell surface staining of AXL and EPHA2 in (D) parental and (E) ADRN-to-MES transition cell line models. Flow cytometry figures are comparing unstained (lighter) to stained (darker) for each sample.

In the ADRN-dominant patient samples and cell lines, we observed subtype-specific RNA expression of candidate immunotherapeutic targets, including *ALK, GPC2*, and *DLL3*, as well as the targeted radiotherapeutic target *SLC6A2* (NET) (Figure 3A, Supplementary Figure 6A and B), including subtype-specific deposition of enhancer histone marks at each gene locus (Supplementary Figure 6C). St. Jude’s Human Tumor Atlas Project supported (Supplementary Figure 7A and B) higher expression of ALK in ADRN and sympathoblast clusters and similar expression of SLC6A2 across ADRN/MES subtypes. We did not observe differential DNA methylation patterns at select subtype-specific loci between ADRN (*N* = 120) and MES (*N* = 8) samples (Supplementary Figure 6D and E). We also observed ADRN-specific expression of ALK at the protein level (Figure 3B). We validated these findings by showing that ADRN-to-MES transdifferentiation results in a loss of ADRN-specific target gene expression of these therapeutically tractable proteins (Figure 3A and C and Supplementary Figure 6A and B). In the KPNYN model with a complete subtype switch to MES, expression of *ALK*, *DLL3*, *GPC2*, and *SLC6A2* were substantially decreased to complete or near-complete loss. In the SKNBE2 cell line with gain of MES signature and retention of ADRN signature, the expression of these therapeutic target genes decreased partially or did not change. Overall, these data suggest that these clinical and preclinical cell surface therapeutic targets are enriched in ADRN-predominant cells and are downregulated in MES-predominant cells.

In contrast, *CD276* and *L1CAM* were similarly expressed across both MES and ADRN subtypes (Figure 3A). The Human Tumor Atlas project suggested pan-subtype expression with slightly higher expression in MES cells (Supplementary Figure 7C). We supported these findings by showing pan-subtype enhancer marks and protein expression across a panel of neuroblastoma cell line models (Figure 3B, Supplementary Figure 6C). Further, *CD276* mRNA and protein expression increased or stayed the same in both KPNYN and SKNBE2 transdifferentiation models assayed, while *L1CAM* showed discordant results (Figure 3A and C). Overall, these data support B7-H3 and L1CAM as subtype-independent neuroblastoma immunotherapeutic targets.

We assessed MES-specific genes that were present in the Pediatric Relevant Molecular Target List to enrich for proteins that are recognized as potential therapeutic targets across childhood cancers.^46,60^ We showed that the receptor tyrosine kinases (RTKs) *AXL*, *EGFR*, *PDGFRA*, *PDGFRB*, and *EPHA2* were specifically enriched in MES-dominant patient samples, as well as MES neuroblastoma cell lines (Figure 3A). Single-cell data from the Human Tumor Atlas supported MES-dominant expression in all the above targets, excepting EPHA2 (Supplementary Figure 7D). We evaluated the expression of AXL in various single-cell (and single-nucleus) datasets and also detected expression in the tumor microenvironment, especially in MES, immune, and schwann cell compartments (Supplementary Figure 8). We observed H3K27ac enhancer marks at the loci of most MES-specific RTKs in the MES neuroblastoma cell line, while ADRN models displayed H3K27me3 marks (Supplementary Figure 6C). This effect was particularly pronounced at the *AXL* and *EPHA2* loci. At the protein level, AXL, EPHA2, and PDGFRB showed MES-specific expression (Figure 3D). Despite some variation at the level of histone marks, transition from ADRN-to-MES resulted in significant upregulation of MES-specific RTK transcripts (Figure 3A), as well as protein measured by immunoblotting and flow cytometry (Figures 3C and E and Supplementary Figure 6F).

### MES-Specific RTKs Are Therapeutic Vulnerabilities in MES Neuroblastoma Cell Line Models

AXL is linked to the EMT gene signature, and by altering the cell signaling pathways, AXL and other RTKs may confer resistance to chemotherapy and targeted therapies.^61–63^ We sought to further understand AXL and its expression on tumor and stromal cells with our deconvolution algorithm. As AXL was minimally expressed in the ADRN subtype, we did not observe any correlations between AXL expression and tumor/immune cell fraction in ADRN-dominant samples (Supplementary Figure 9). We did observe a negative correlation between AXL expression and tumor fraction in MES-dominant samples (cor = −0.5; *P* = 6.85e-3) (Supplementary Figure 9A), though the MES-dominant samples with the highest tumor purity still expressed AXL at higher levels than ADRN-dominant samples. We showed that AXL expression is positively correlated with monocytes in the MES-dominant tumor samples (cor = 0.48; *P* = 1.04e-2; Supplementary Figure 9B). Together, these data and our in vitro data (Figure 3) suggest that AXL is present in both neuroblastoma cells and cell populations within the tumor microenvironment.

To assess the functional role of AXL on tumor cells, we modulated its expression in neuroblastoma cell line models and monitored the impact on subtype-specific markers. After doxycycline-inducible *AXL* overexpression in neuroblastoma cell lines, we observed subtle changes in the IMR5 model whereby some MES markers slightly increased and some ADRN markers slightly decreased (Supplementary Figure 10A–C), supporting AXL as a contributor to the MES subtype. After complete *AXL* knockout using CRISPR/Cas9, we did not observe changes in MES or ADRN markers (Supplementary Figure 10D–F). These data suggest that AXL is not solely necessary for maintenance of the MES subtype but likely contributes in combination with other signaling proteins.

We next evaluated the activity of several MES-specific RTK small molecule inhibitors in development. For AXL, we examined the cytotoxicity of bemcentinib, cabozantinib, NPS-1034, and ONO-7475 in a panel of ADRN and MES neuroblastoma cell lines. While bemcentinib was similarly cytotoxic in both ADRN and MES models, the remaining AXL inhibitors were slightly more active in MES models (Supplementary Figure 11A). Despite increased cytotoxicity in MES models, the drugs were not especially potent, as the minimum IC50 values for cabozantinib, NPS-1034, and ONO-7475 were 1.1 µM, 876 nM, and 144 nM, respectively (Supplementary Figure 11C). Additionally, depletion of AXL did not result in resistance to AXL inhibition (Supplementary Figure 12), suggesting that cytotoxicity was mediated in part by nonspecific inhibition of other RTKs. The EPHA2 inhibitor ALW II-41-27 and the multitarget inhibitor dasatinib showed MES-specific activity across a panel of parental neuroblastoma cell lines (Supplementary Figure 11B). For the EGFR inhibitors erlotinib and sapitinib, we observed that MES models were slightly more sensitive compared to ADRN-like models (Supplementary Figure 13A), whereas PDGFR inhibition did not show subtype-specific cytotoxicity (Supplementary Figure 13B).

### AXL-Specific ADG (ADCT-601) Shows Antitumor Activity

**Figure 4.**
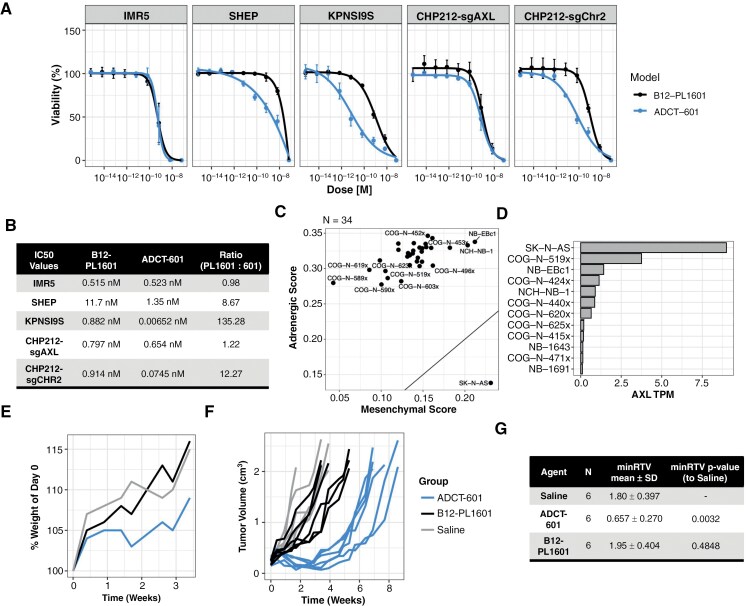
AXL-specific antibody–drug conjugate (ADCT-601) demonstrates activity in vitro and in vivo. (A) Cellular viability and (B) IC50 values after 5 days of treatment with Control ADC (B12-PL1601) or AXL-targeted ADC (ADCT-601). Data were collected from 3 independent experiments. Ratio reported is B12-PL1601:ADCT-601. (C) Adrenergic (ADRN)/mesenchymal (MES) signature scores and (D) AXL mRNA expression in patient-derived and cell line-derived xenograft models. (E) Mouse weights after treatment (Day 0) as a percent of Day 0. (F) Tumor volumes after treatment (Day 0) of individual mice. Treatments are Saline, Control ADC (B12-PL1601, 1 mg/kg dose), or AXL-targeted ADC (ADCT-601, 1 mg/kg dose). *N* = 6 mice per treatment arm. (G) Minimum relative tumor volume (minRTV) mean and standard deviation (SD) per treatment group with associated statistics using pairwise Wilcoxon rank-sum test.

Because we posit that AXL has both tumor cell intrinsic and extrinsic functions in mediating a MES cell state, and it appears that AXL signaling alone is not a major oncogenic drive of the MES cell state, we explored ADCT-601, an AXL-targeted ADC, which contains a potent pyrrolobenzodiazepine dimer toxin to explore MES-specific therapeutic targeting. We evaluated the activity of ADCT-601 and isotype control ADC B12-PL1601 (targeting HIV envelop protein gp120) across a panel of neuroblastoma cell lines. In an ADRN model (IMR5) and a MES-derived model with *AXL* genetically deleted (CHP212-sgAXL), we observed no difference between the ADCT-601 and B12-PL1601 (Figure 4A). Conversely, MES-like models with cell surface AXL (Figures 3D and Supplementary Figure 10D and E) were more sensitive to ADCT-601 compared to B12-PL1601 (Figure 4A). We calculated ADCT-601 IC50 values of 6.52 pM in KPNSI9s and 74.5 nM in CRISPR control model CHP212-sgCHR2 (Figure 4B). The MES model SHEP was generally less sensitive to these agents (IC50s: ADCT-601 at 1.35 nM and B12-PL1601 at 11.7 nM). Our observed IC50 values for ADCT-601 are within previously published ranges (20 pM–2.2 nM).^54^

We next extended our in vitro studies to PDX or CDX models. Only 1 PDX or CDX, SKNAS, was MES-dominant and expressed *AXL* (Figure 4C and D). In vivo studies with SKNAS (*N* = 6/arm) and the ADRN COGN519x PDX (*N* = 3/arm) show that a single dose of ADCT-601 at 1 mg/kg was well-tolerated (Figure 4E, Supplementary Figure 14A). In SKNAS-bearing animals, we observed a significant decrease in minimum relative tumor volume (minRTV) compared to the saline control (*P* = 0.0032; Figure 4F and G). Antitumor activity with ADCT-601 was not seen in the ADRN COGN519x model (Supplementary Figure 14B). Altogether, our data support the further development of AXL-targeted ADCs in MES-dominant neuroblastoma models.

## Discussion

Neuroblastoma is a heterogeneous disease in terms of clinical manifestations, natural history, and response to therapy. While somatic genomic heterogeneity explains in part clinical diversity and therapy resistance,^64,65^ it has been known for almost 50 years that neuroblastoma cells in culture can assume vastly different cell states.^26^ More recently, the epigenetic basis of this observation has been defined,^28,29,66^ and recent data suggest that relapsed tumors are enriched in MES gene signatures.^63^ Here, we characterized patient tumors by their dominant ADRN or MES signature to evaluate the expression of proven and candidate therapeutic targets in the admixed cells that reside in neuroblastomas. We extended prior observations that the MES subtype is more immunogenic by observing an association with the inflammatory response.^56,57^ These genes also show subtype-specific histone marks at these loci, and forced transdifferentiation from ADRN to MES is associated with significantly increased expression. This suggests that MES cells may be vulnerable to immunotherapeutic approaches, perhaps targeting cancer-specific peptides presented on common HLA allotypes,^67^ or cell surface proteins enriched in this state. It is notable, however, that the expression of HLA-E, which inhibits NK and CD8^+^ cytotoxic T cells,^68,69^ is also increased in MES cells and may confer immune evasion. Whether blocking HLA-E:NKG2A interaction with specific antibodies^70,71^ renders the MES cells more susceptible to immunotherapy remains to be established.

Our study underscores the importance of neuroblastoma subtype heterogeneity and its implications on targeted therapies. We show that multiple immunotherapeutic targets in clinical development are ADRN-predominant and are expressed at significantly lower levels, or even absent, in MES-like tumors and cell lines. These findings are supported by ADRN-to-MES transdifferentiation experiments in cell line models and evaluation of the epigenome in parental cell lines. In contrast, *CD276* and *L1CAM* show relatively stable expression in ADRN and MES cells. In our recent preclinical study using a CD276-targeted ADC (m276-SL-PBD), we observed an objective response rate of 93% in the neuroblastoma CDXs and PDXs.^19^ Importantly, all neuroblastoma PDXs assessed display a strong ADRN signature, so the efficacy of CD276-targeted agents, as well as other preclinical molecules, remains to be determined in MES-dominant neuroblastomas. While antigen loss is a known mechanisms of escape from many immunotherapies, our data suggest that this might largely occur by epigenetically mediated cell state transitions in neuroblastoma, and this needs to be considered in ongoing and future clinical trials.

We identified several candidate MES-specific RTKs, including *AXL*, *EPHA2*, *EGFR*, and *PDGFRA/B*. Several of these genes have been described as important mediators of cancer cell resistance to targeted therapies.^61–63,72^ Recently, Noronha et al. described increased signaling through the GAS6-AXL signaling axis in drug-tolerant persister cells, which can mediate resistance to RTK inhibitors in *EGFR*-mutant lung cancer models.^73^ We showed enrichment of these proteins in MES cell lines and observed upregulation of these genes after ADRN-to-MES transdifferentiation. However, despite AXL being a DEG in MES-dominant tumor samples and cell line models, here we show that it is neither necessary for MES subtype maintenance nor sufficient to transdifferentiate ADRN cells to a MES-like phenotype. Thus, the potential role of AXL RTK inhibitors to target MES cell state in this disease requires further exploration, but it is of interest that the multi-RTK inhibitor dasatinib has been used in a metronomic regimen for disease palliation.^74^

Finally, we assessed the activity of ADCT-601, a pyrrolobenzodiazepine dimer-based AXL-targeted ADC, in neuroblastoma cell lines and CDX/PDX models. We show that a single dose of ADCT-601 is well-tolerated and demonstrates significant antitumor activity in a MES CDX model. AXL-targeted ADCs have been evaluated in non-small-cell lung cancer,^75^ soft tissue sarcoma,^76^ and melanoma,^77^ in combination with small molecule inhibitors or immune checkpoint blockade,^78^ and Phase I clinical trials with ADCT-601 are ongoing (NCT05389462). As a pan-cancer MES marker, AXL upregulation can be observed after treatment with targeted therapy.^63^ Thus, combination strategies with AXL-targeted ADCs aim to prevent resistance by targeting therapy-induced heterogeneity.^77^ In experimental neuroblastoma models, AXL has been reported to contribute to therapy resistance to ALK inhibitors.^62^ Future studies evaluating various treatment schedules of ADCT-601 in additional MES-dominant models will be essential to further determine the efficacy of this compound. Additionally, studies incorporating heterogenous tumors with combination strategies will be necessary to credential AXL as an effective therapy for the elimination of MES-specific neuroblastoma tumor cells and/or the treatment-resistant neuroblastomas.

As most high-risk neuroblastomas are ADRN-dominant, successful clinical developments have focused on targeting molecules abundant in this subtype. However, studies investigating neuroblastoma heterogeneity show the plasticity of neuroblastoma cells and consequently, the ADRN-specific molecules of clinical interest. Therefore, it is critical to further profile these neuroblastoma cell subtypes with single-cell sequencing technologies, generate in vivo models that are representative of the MES subtype, and develop approaches to target the cells that can persist ADRN-targeted modalities. As more targeted therapies are brought forward to the clinic, serial monitoring of tumor specimens will be essential to understand the temporal heterogeneity in relation to treatment response and allow clinicians to identify and treat emerging resistance.

## Supplementary Material

Supplementary material is available online at *Neuro-Oncology* (https://academic.oup.com/neuro-oncology).

noaf012_suppl_Supplementary_Materials

## Data Availability

Code and data produced will be made available upon reasonable request.
