# Supplementary material for: Lineage dependence of the neuroblastoma surfaceome defines tumor cell state-dependent and -independent immunotherapeutic targets
Source: Neuro Oncol. 2025 Jan 18;27(5):1372–84. doi: 10.1093/neuonc/noaf012 (PMC12187457; doi:10.1093/neuonc/noaf012)
Supplement: noaf012_suppl_Supplementary_Materials [file noaf012_suppl_supplementary_materials.docx]

**Supplemental Methods and Figures**

*RNA-sequencing Data*

Neuroblastoma RNA sequencing data was accessed through the Treehouse Childhood Cancer Initiative and Gabriella Miller Kids First (GMKF) Pediatric Research Program. Treehouse data was obtained as expected count matrices aligned to Hg38 (Treehouse Tumor Compendium v11 Public PolyA <https://treehousegenomics.soe.ucsc.edu/public-data/#tumor_v11_polyA>, N = 200 tumors). GMKF data was collected from the GMKF Data Resource Center (<https://d3b.center/kidsfirst/>, N = 195 samples). We only assessed samples that were confirmed as neuroblastoma by histopathology. Thirteen patient samples were represented in both the Treehouse and GMKF datasets. For differential expression analysis, Treehouse and GTEx datasets were converted to counts per million (cpm), filtered by genes with cpm>1, and normalized with the Empirical Analysis of Digital Gene Expression Data package (edgeR) (trimmed mean of M-values, or TMM) for downstream analyses.^1,2^ We also utilized our RNA-sequencing data for parental neuroblastoma cell lines (N=38) and patient-derived xenografts (N=30) as previously described.^3,4^ RNA-sequencing for ADRN-to-MES transition models (SKNBE2 and KPNYN) was obtained from the NCBI Gene Expression Omnibus [GSE180516].^5^ Single-cell RNA-sequencing datasets were collected from published studies,^6-9^ and processed using our methods as described.^8^

*Subtype Classification*

Neuroblastoma samples (Treehouse human tumors, GMKF human tumors, and neuroblastoma cell lines) were analyzed with singscore, a single sample gene set enrichment analysis (ssGSEA) method.^10^ The gene set lists for ADRN and MES cell states were obtained from Groningen and colleagues.^11^

*Differential Expression (limma)*

Log2TPM RNA-seq data were transformed with the *voom* function and modeled with lmfit.^12^ Contrasts were prepared between each neuroblastoma subtype. For each comparison, the linear model was evaluated with limma using empirical Bayes Statistics (eBayes) and adjustment with Benjamin-Hochberg post hoc testing.^13^ To evaluate ADRN-specific and MES-specific differentially expressed genes, we filtered by a minimum Log2 fold change >1 and max adjusted p value <0.05. Pan-subtype genes were defined by a minimum Log2 fold change <1 and max adjusted p value >0.05. Surface proteins were annotated with the UniProt localization database with the keyword “Cell Membrane”. Subtype-specific and pan-subtype genes were also intersected with the Food and Drug Administration’s Pediatric Relevant Molecular Target List for childhood cancers (<https://www.fda.gov/media/120332/download>).^14^

*Deconvolution*

Two algorithms were used to predict cellular fractions of bulk patient RNA-sequencing data. quanTIseq was performed with bulk TPM data and the --tumor=TRUE flag**.**^15^ EPIC was performed with bulk TPM data after removing common genes in both MES-specific neuroblastoma and cancer-associated fibroblast signatures (COL1A1, COL3A1, SYNPO2).^16^

*ChIP-sequencing Analysis*

ChIP-sequencing data for H3K4me1, H3K4me3, H3K27Ac, and H3K27me3 were obtained from the GEO database (GSE138315).^17^.

*DNA-methylation Analysis*

Methyl-sequencing data were obtained from the TARGET project available on the NCI Office of Cancer Genomics website (<https://target-data.nci.nih.gov/Public/NBL/methylation_array/>).^18^ Samples with available mRNA-sequencing data were used to determine dominant subtype (120 ADRN samples and 8 MES samples). The R package gviz was used to generate figures.

*GSEA*

The *fgsea* and *msigdbr* packages were utilized to find gene sets associated with both ADRN and MES Treehouse and GMKF patient tumors.^19,20^

*Cell Culture*

Neuroblastoma cell lines were cultured in RPMI 1640 Medium with L-glutamine (Corning cat. 10041CV) with 10% FBS (VWR cat. 89510-186) and 1% L-glutamine (Corning cat. 25005CI) or IMDM (ThermoFisher cat. 12440053) with 20% FBS, 1% L-glut, and 1:1000 ITS+ Premix Universal Culture Supplement (Corning cat. 354352). When culturing cell lines with doxycycline-regulated transgenes, we used Tet-Free FBS (Takara cat. 631106). Transdifferentiation models (KPNYN and SKNBE2) were detailed in co-authors’ publication^5^. Briefly, a PRRX1 gBlock was cloned into pINDUCER20 (Addgene cat. 44012) via Gateway cloning and further prepared into lentiviral particles for inducible overexpression in transduced cell models. These models were exposed to doxycycline (500mg/µL) for at least 21 days to establish stably differentiated cells prior to experimentation. Additional transdifferentiation models (SKNBE2C-PRRX1 and IMR5-ICN3) were detailed in co-authors’ publication^21^. Intracellular NOTCH3 (ICN3) in the pTRIPZ vector was a gift from Dr. Hiroshi Nakagawa. It was cloned into pLVX-TetOne (Takara cat. 631847) and used as previously described for this vector.

For CRISPR/Cas9 knockout of *AXL*, we utilized the lentiCRISPRv2 vector (Addgene cat. 52961) with one of two *AXL*-targeted sgRNAs (Oligo 1: CACCGCTGAGAACATTAGTGCTACG; Oligo 2: CACCGGCTGCTGGTGCATGCCACG) ^22^. We also cloned a non-targeting (sgLacz: CACCGAACGGCGGATTGACCGTAAT) and an off target sgRNAs (Chr2.2: CACCGGTGTGCG-TATGAAGCAGTG). lentiCRISPRv2 constructs were independently packaged into lentiviral particles in HEK293T cells with psPAX2 (Addgene cat. 12260) and pMD2.G (Addgene cat. 12259), according to the Lipofectamine 2000 protocol (Invitrogen cat. 11668027). Cell line models were transduced with lentiviral particles and polybrene (10µg/mL) containing one of four lentCRISPRv2 constructs and selected with puromycin (1µg/mL) for several weeks.

AXL overexpression was achieved with the TetR protein and a CMV/TO-regulated *AXL* transgene. We used Gateway Clonase II (Invitrogen cat. 11791020) to insert the *AXL* cDNA (GeneCopoeia cat. GC-Z7835) into pLenti-CMV/TO-puroR-DEST (Addgene cat. 17293). To regulate expression of the CMV/TO-AXL transcript, we utilized the pLenti-CMV-TetR-blastR construct (Addgene cat. 17492). Both pLenti-CMV/TO-AXL-puroR and pLenti-CMV-TetR-blastR constructs were independently packaged into lentiviral particles, as above. Parental neuroblastoma cell lines were successively transduced with the TetR-containing lentivirus, selected with 5µg/mL blasticidin (Invitrogen cat. R21001), transduced with the CMV/TO-AXL lentivirus, and finally selected with 1µg/mL puromcyin (Gibco cat. A1113803). Expression of *AXL* was induced by adding 1µg/mL of doxycycline (Sigma cat. D3072) to the culture medium.

*Sample Processing and Immunoblotting*

Whole-cell lysates were prepared using RIPA Lysis Buffer System (Santa Cruz Biotechnology cat. sc-24948), Protease Inhibitor Cocktail (Sigma-Aldrich cat. P8340), and Phosphatase Inhibitor Cocktails 2 and 3 (Sigma-Aldrich cat. P5726 and cat. P0044). Pellets were resuspended in lysis buffer, incubated on ice for 15 minutes, disturbed with a vortex for 20 seconds, and then cleared by centrifugation at max speed (17900 x g) for 15 minutes at 4^o^C. Protein concentration was determined according to the Bio-Rad Protein Assay Kit II (Bio-Rad cat. 5000006). Once quantified, 15µg of each protein sample was prepared with Laemmli Sample Buffer (Bio-Rad cat. 1610737) and 50mM DTT (Millipore Sigma cat. 43816). Samples were loaded on a 4-15% Criterion TGX Protein Gel (Bio-Rad cat. 5671085) and run with Tris-Glycine Buffer (Bio-Rad cat. 1610771) containing 0.1% SDS (Invitrogen cat. 15553027) at 40mAmps per gel. Proteins were transferred to a 0.45µm Immobilon-P Membrane (Millipore Sigma cat. IPVH00010) in ice-cold Tris-Glycine Buffer with 20% methanol in a transfer tank either overnight at 10V or 1 hour at 50V. Membranes were blocked with 5% Blotting-Grade Blocker (Bio-Rad cat. 1706404) in Tris Buffered Saline with Tween® 20 (TBST, Cell Signaling Technology cat. 9997) for 1 hour and incubated with primary antibody in 5% Blocking Buffer overnight at 4^o^C. Primary Antibodies and dilutions are as follows: ALK (1:1000, CST cat. 3333), DLL3 (1:1000, CST cat. 78110), GPC2 (1:500, SCBT cat. Sc-393824), SLC6A2 (1:500, MAb Technologies cat. NET17-1), CD276 (1:1000, Abcam cat. ab134161), L1CAM (1:1000, CST cat. 89861), AXL (1:200, R&D Systems cat. AF154), EGFR (1:1000, Thermo Fisher cat. MA5-13070), EPHA2 (1:1000, Thermo Fisher cat. 37-4400), PDGFRA (1:1000, Thermo Fisher cat. 710169), PDGFRB (1:1000, Thermo Fisher cat. MA5-15143), PHOX2A (1:1000, SCBT cat. sc-81978), DBH (1:1000, CST cat. 8586), SNAI2 (1:1000, CST cat. 9585), VIM (1:1000, CST cat. 5741). Membranes were then washed 4 times for 10 minutes with TBST. We then incubated membranes with secondary antibody (in 5% Blocking Buffer) for 1 hour at room temperature (Invitrogen cat. A16110; Invitrogen cat. 31432; Invitrogen cat. 31402; R&D Biosystems cat. HAF016). We performed a final TBST wash (4 x 10 minutes) before incubating membranes in Pierce ECL Plus (Thermo Scientific cat. 32132) or SuperSignal West Femto ECL (Thermo Scientific cat. 34095) for 5 seconds to 3 minutes. We imaged membranes using the Azure Biosystems Sapphire^TM^ Biomolecular Imager.

*Flow Cytometry*

Neuroblastoma cell lines were counted and distributed into FACS tubes for various staining conditions and an unstained control (0.5M cells / condition). Cells were washed 3 times with 1mL of PBS, spinning at 300 x g for 5 minutes at 4^o^C between each step. Staining solutions were prepared in PBS with the manufacturer’s recommended antibody and LIVE/DEAD Fixable stain (Invitrogen cat. L34980) amounts per assay. Conjugated primary antibodies include the following: APC-AXL (R&D Systems cat. FAB154A), PE-EphA2 (BioLegend cat. 356803), APC-PDGFRA (BioLegend cat. 323511), AF488-HER2 (BioLegend cat. 324410), BV421-EGFR (BioLegend cat. 352911), BV605-PDGFRB (BD Biosciences cat. 743035). Cells were resuspended in 100µL of the appropriate staining buffer and incubated on ice and in the dark for 30 minutes. After the antibody incubation, cells were again washed 3 times with 1mL of PBS, spinning at 300 x g for 5 minutes at 4^o^C between each step. Cells were fixed in 1% formaldehyde ice and in the dark for 15-30 minutes, then washed 2 times with 1mL of PBS. Finally, cells were resuspended in 100µL of PBS and stored at 4^o^C in the dark until samples were analyzed on a CytoFLEX LX Flow Cytometer (Beckman Coulter). Data was analyzed using FlowJo® software.

*Cytotoxicity Assays*

Small molecule inhibitors were obtained from Selleck Chemicals: Bemcentinib (S2841), Cabozantinib (S1119), NPS-1034 (S7669), ONO-7475 (S8933), Erlotinib (S7786), Crenolanib (S2730), Afatinib (S1011), Imatinib (S2475), Sitravatinib (S8573), Dasatinib (S1021), Erdafitinib (S8401), ALW II-41-27 (S6515), Sapitinib (S2192). AXL-targeting (ADCT-601) and control (B12-PL1601) antibody-drug conjugates (ADCs) were studied in collaboration with ADC Therapeutics ^23^. Neuroblastoma cell lines were seeded in a 96-well plate one day prior to treatment with small molecule inhibitors or antibody-drug conjugates. Assays with small molecule inhibitors (128pM – 50µM) were concluded after 96 hours, while ADCs studies with (0.667fM – 66.7nM) were evaluated after 120 hours. At the study endpoint, cellular viability was determined using the CellTiter-Glo 2.0 Assay protocol (Promega cat. G9243). Luminescence values were normalized to untreated wells and data was analyzed using R 4.0.3 (2020-10-10). Plots and IC50 values were calculated using the following packges: ggplot2 (v3.3.5), nplr (v0.1-7), drc (v3.0-1), dr4pl (v2.0.0), tidyverse (v1.3.1). Each experiment was plated in technical triplicate and data are representative of at least two independent experiments.

*In Vivo Studies*

For murine efficacy studies, we engrafted CB17 severe combined immunodeficiency (SCID) mice with cell line-derived xenografts (CDXs) or patient-derived xenografts (PDXs) and followed the standard protocol established in the Pediatric Preclinical Testing Consortium (now named Pediatric Preclinical In Vivo Testing, PIVOT).^24^ In brief, mice with tumors at enrollment size (0.2-0.3 cm^3^) received 1 mg/kg of either ADCT-601 or B12-PL1601 via tail vein injection. Studies were performed with N=6 animals per arm and the mice were monitored for 100 days or until their tumor burden reached 2.0 cm^3^.

| **S. Figure 1. ADRN- and MES-dominant patient samples and cell lines are characterized by differentially expressed genes driven by subtype-specific enhancers.** |
| --- |
| 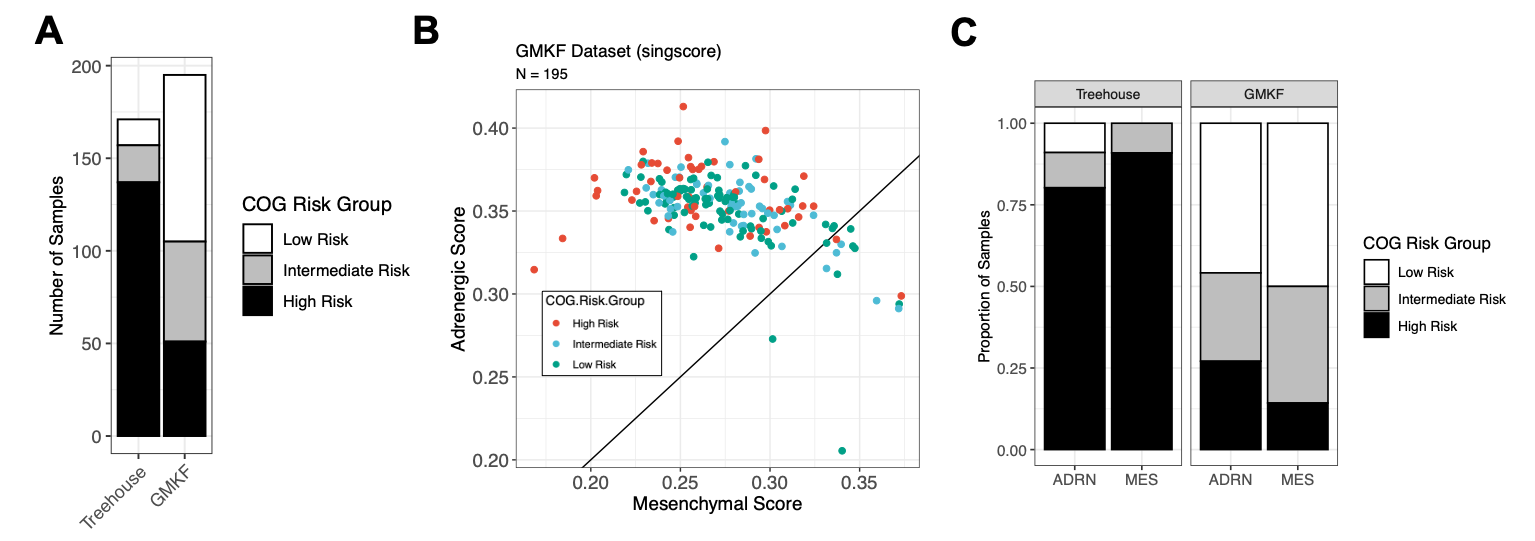  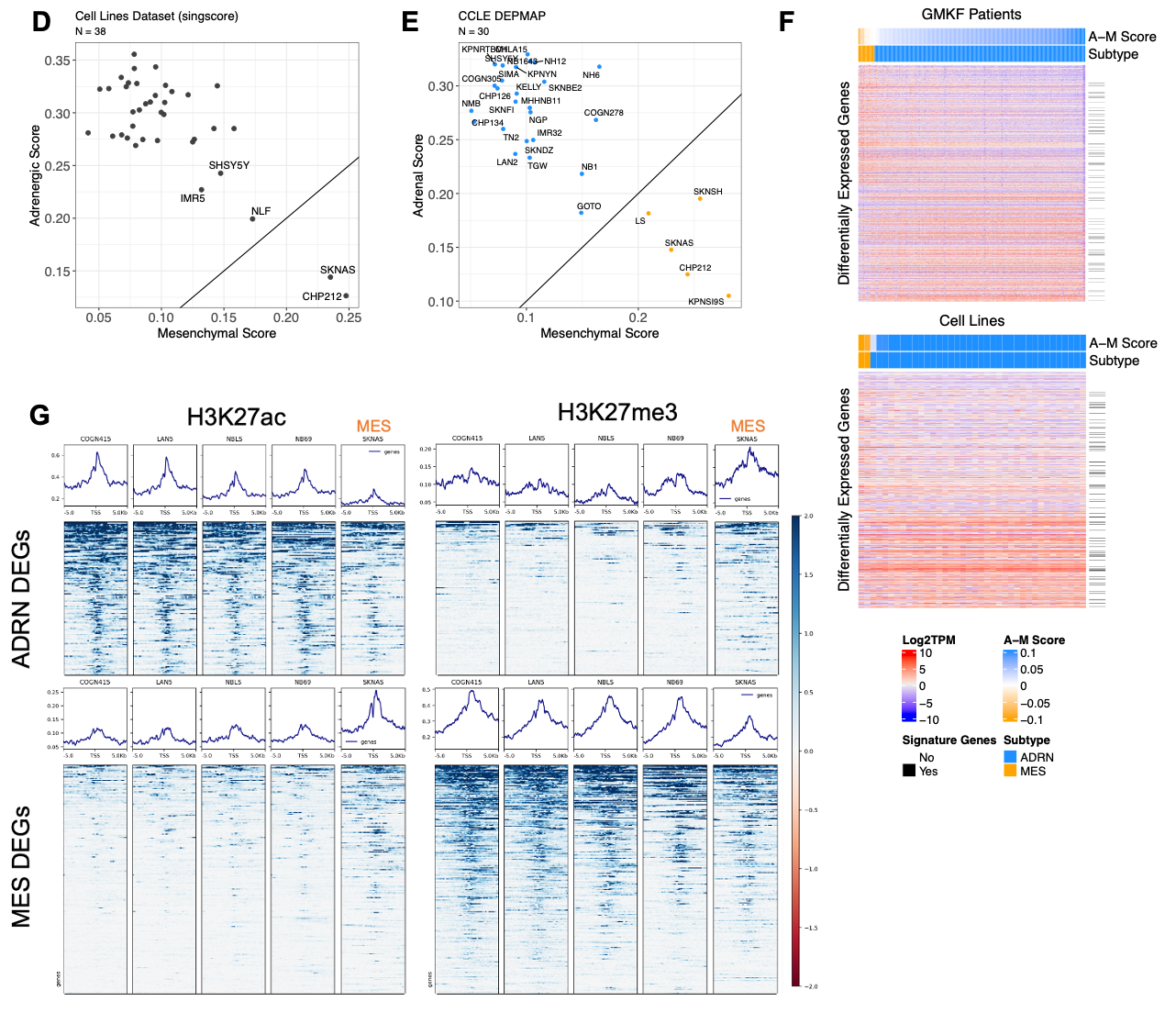 |
| S. Figure 1 Legend: A) Distribution of COG Risk Group across Treehouse and GMKF datasets, (B) subset by a ADRN and MES. C-E) ADRN and MES scores calculated by single-sample gene set enrichment analysis (singscore) in (C) GMKF patient samples and (D-E) neuroblastoma cell line models. E-F) Heatmaps of top 500 MES and ADRN differentially expressed genes (from Treehouse) in (E) GMKF patient samples and (F) neuroblastoma cell line models. G) Histone ChIP-sequencing (H3K27ac or H3K27me3) in ADRN or MES neuroblastoma cell lines at ADRN and MES differentially expressed genes. |

| **S. Figure 2. Pathway enrichment in MES- and ADRN-dominant patient samples and cell lines.** |
| --- |
| 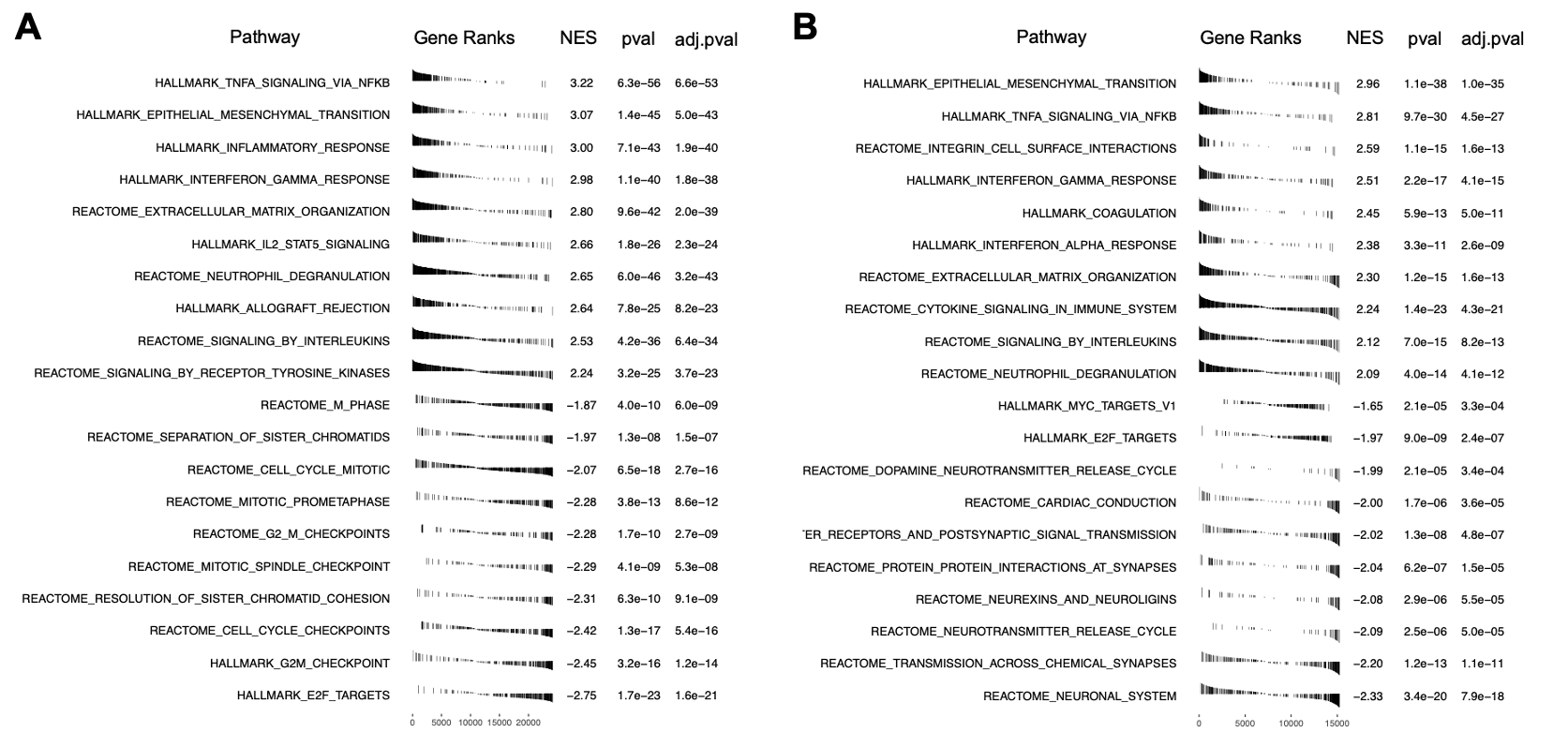 |
| S. Figure 2 Legend: A-B) Gene set enrichment analysis (fgsea) of MES- and ADRN-dominant (A) Treehouse patient samples and (B) ADRN-to-MES transdifferentiation models using Hallmark and curated (C2) MSigDB pathways. Values represent normalized enrichment scores (NES), p-values (pval, based on multi-level split Monte-Carlo scheme), and Benjamini-Hochberg-adjusted p-values (adj.pval). |

| **Supplemental Figure 3. Estimated tumor and immune cell fractions in bulk RNA-sequencing datasets.** |
| --- |
| 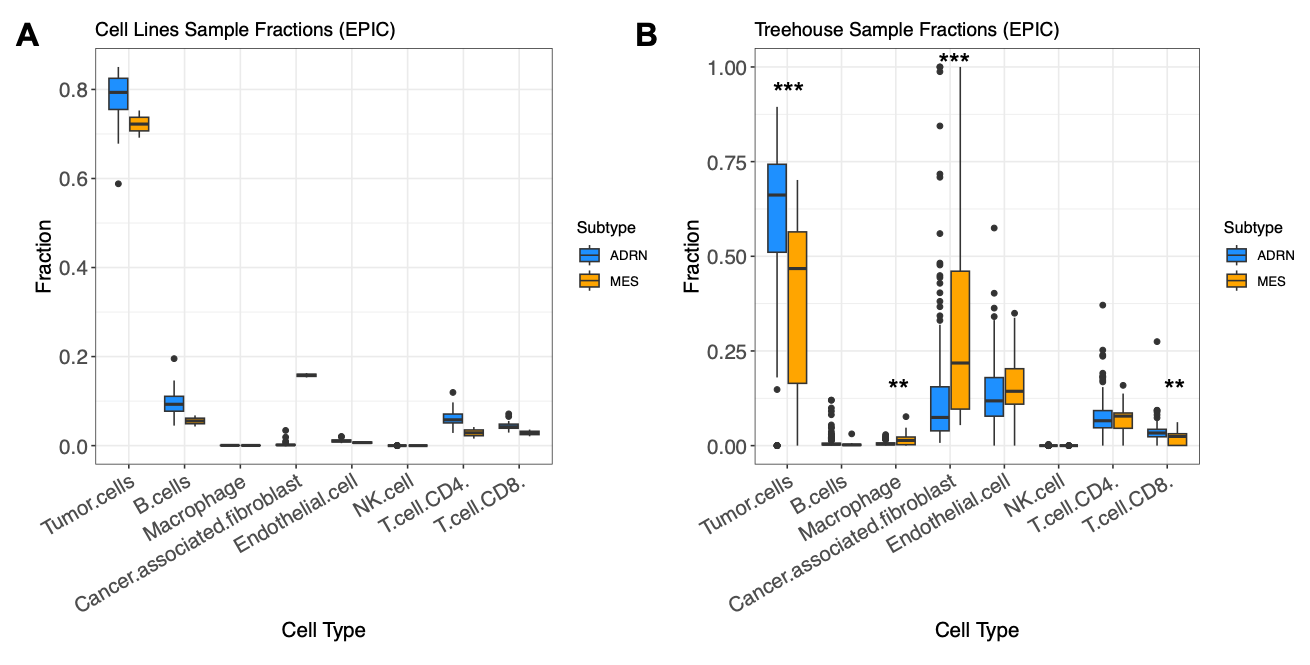 |
| S. Figure 3 Legend: A-B) Tumor and immune cell fractions predicted with deconvolution algorithm EPIC in (A) neuroblastoma cell lines and (B) Treehouse patient bulk RNA-sequencing datasets. Statistics are as follows: * p<0.5, ** p<0.01, *** p<0.001. |

| **Supplemental Figure 4. Epigenetic regulation of MHC processing/presentation genes and immunomodulatory genes.** |
| --- |
| 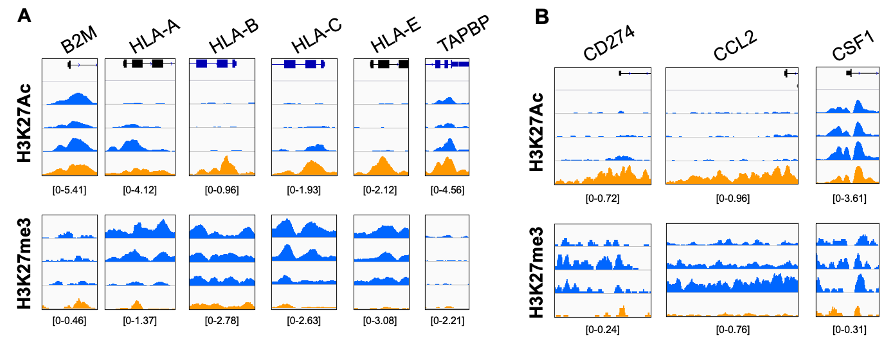 |
| S. Figure 4 Legend: A-B) Histone ChIP-sequencing peaks at target loci representing H3K27ac (enhancer, top) and H3K27me3 (repressive, bottom) marks in COGN415, KELLY, SKNBE2C, and SKNAS models. Colors represent ADRN (blue) and MES (orange) cell lines. |

| **Supplemental Figure 5. MYC(N) expression and epigenetic marks across neuroblastoma cell subtypes.**  **** |
| --- |
|  |
| S. Figure 5 Legend: A-B) RNA expression of (A) MYCN and MYC in Treehouse Patient samples, cell lines, and SKNBE2 and KPNYN before (parent) and after (PRRX1) transdifferentiation. Statistics represent Wilcoxon test using compare_means() function in R. C) Histone ChIP-sequencing peaks at target loci representing H3K27ac (enhancer, top) and H3K27me3 (repressive, bottom) marks in COGN415, KELLY, SKNBE2C, and SKNAS models. |

| **Supplemental Figure 6. Differential expression and epigenetic regulation of candidate subtype-specific and pan-subtype targets.**  **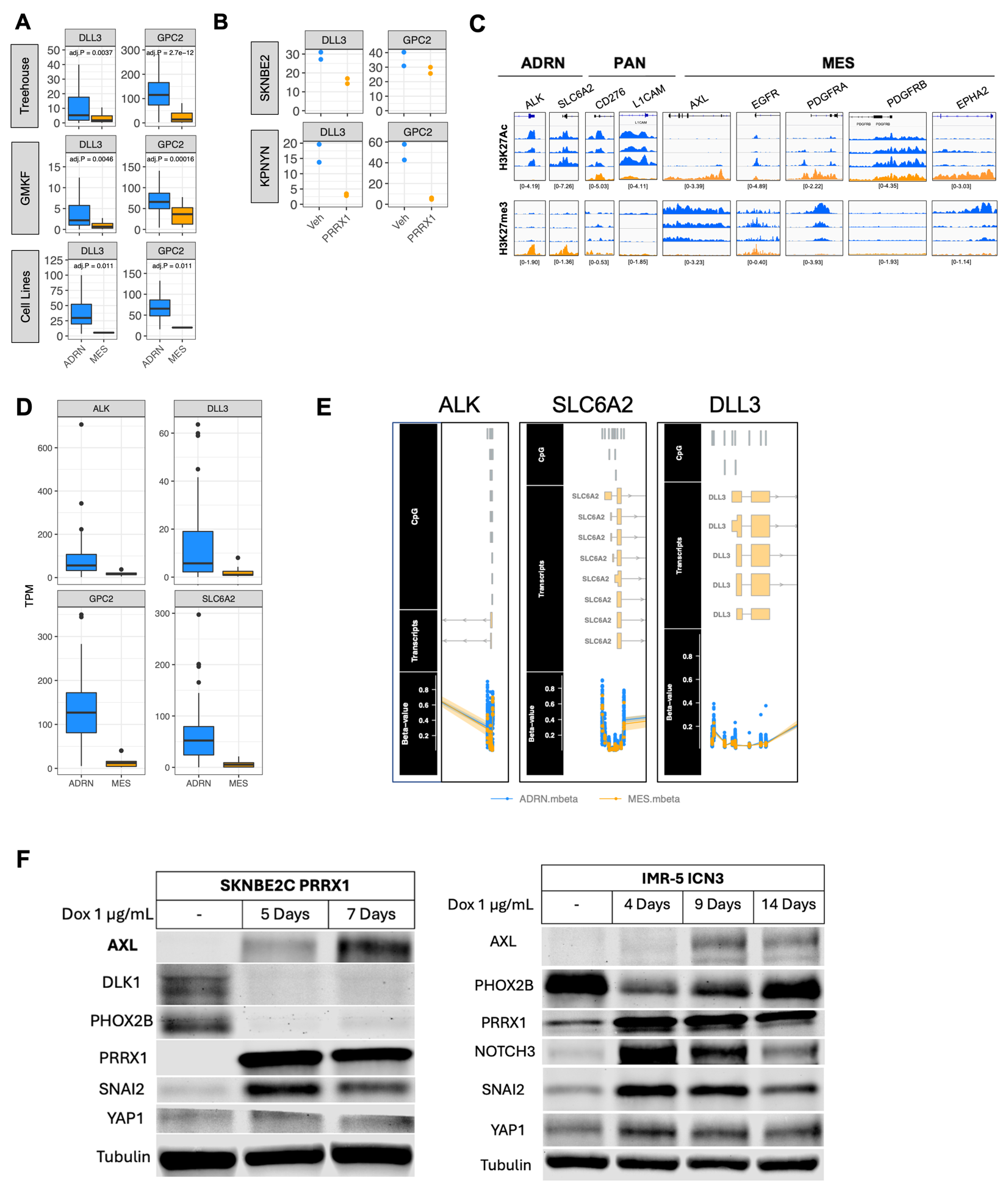** |
| --- |
|  |
| S. Figure 6 Legend: A-B) RNA expression of preclinical ADRN-specific targets, DLL3 and GPC2, in (A) patient datasets, cell lines, and (B) ADRN-to-MES transdifferentiation models. B) Histone ChIP-sequencing peaks at target loci representing H3K27ac (enhancer, top) and H3K27me3 (repressive, bottom) marks in COGN415, KELLY, SKNBE2C, and SKNAS models. D) RNA expression of ADRN-specific targets in subset of patient samples with methylation sequencing data. E) mBeta scores in ADRN-dominant and MES-dominant patients at the ALK, SLC6A2, and DLL3 loci. F) Protein expression of AXL after ADRN-to-MES transdifferentiation in additional neuroblastoma cell lines (SKNBE2C + PRRX1 and IMR5 + ICN3). |

| **Supplemental Figure 7. Expression of neuroblastoma subtype-dominant and pan-subtype targets in Human Tumor Atlas Pilot Project.** |
| --- |
|  |
| S. Figure 7 Legend: A) Annotated clusters of tumor subtypes and tumor microenvironment cell types in Human Tumor Atlas Pilot Project. B-D) Expression violin plots of ADRN-dominant (B), pan-subtype (C), and (D) MES-dominant cell surface targets. Visualization excludes cells without expression. |

| **Supplemental Figure 8. Single-cell datasets support AXL in the tumor and tumor microenvironment.** |
| --- |
|  |
| S. Figure 8 Legend: A-D) Expression of AXL in tumor and tumor microenvironment cell types, (A-C) processed and plotted as in Grossmann et al (Cancer Cell 2024) or (D) in the Human Tumor Atlas Pilot Project. |

| **Supplemental Figure 9. Correlation between predicted fraction (quanTIseq) and AXL mRNA expression.** |
| --- |
| 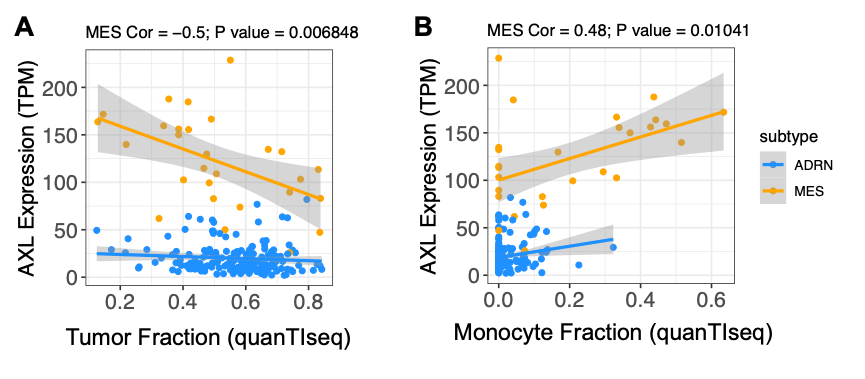 |
| S. Figure 9 Legend: A-B) AXL mRNA expression (TPM) vs. (A) tumor and (B) monocyte fractions predicted by quanTIseq. Spearman correlations presented are only for MES samples. |

| **Supplemental Figure 10. AXL is not sufficient to change, nor necessary to maintain the MES phenotype.** |
| --- |
| 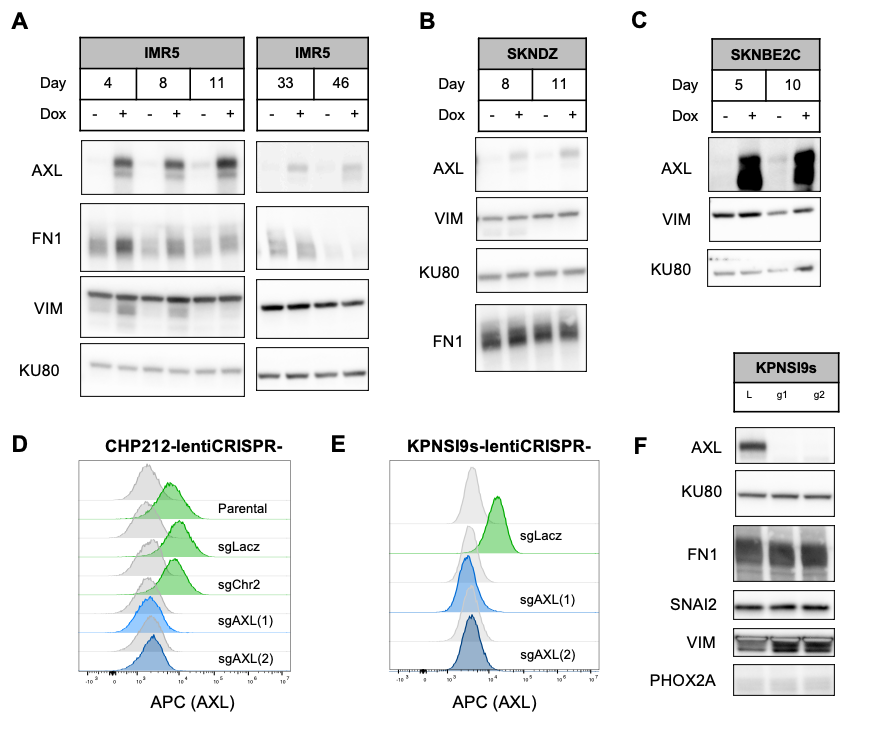 |
| S. Figure 10 Legend: A-C) AXL overexpression was achieved by subsequent transduction of ADRN parental cell lines (IMR5, SKNDZ, SKNBE2C) with lentiviral particles containing the tet repressor protein and then the AXL transgene driven by a CMV/TO promoter. Cells were cultured in the presence of DMSO or doxycycline (1ug/mL) for up to 46 days and assayed for both MES and ADRN markers. D-E) AXL expression measured by flow cytometry after knockout of AXL in the MES models (D) CHP212 and (E) KPNSI9s with the pLentiCRISPRv2 system using two sgRNAs and a control sgRNA targeting LACZ and/or Chr2.2. F) Expression of MES and ADRN markers in KPNSI9s models after AXL knockout. |

| **Supplemental Figure 11. RTK-targeted small molecule inhibitors show specificity towards MES neuroblastoma cell line models.** |
| --- |
|  |
| S. Figure 11 Legend: A-B) Cytotoxicity of small molecule inhibitors targeting **(A)** AXL and **(B)** EPHA2 across a panel of ADRN and MES neuroblastoma cell lines. N represents the number of biological experiments for each inhibitor. Viability was measured with CellTiter-Glo2.0 after 3 days of exposure to each inhibitor. |

| **Supplemental Figure 12. AXL inhibitors do not exclusively target AXL.** |
| --- |
| 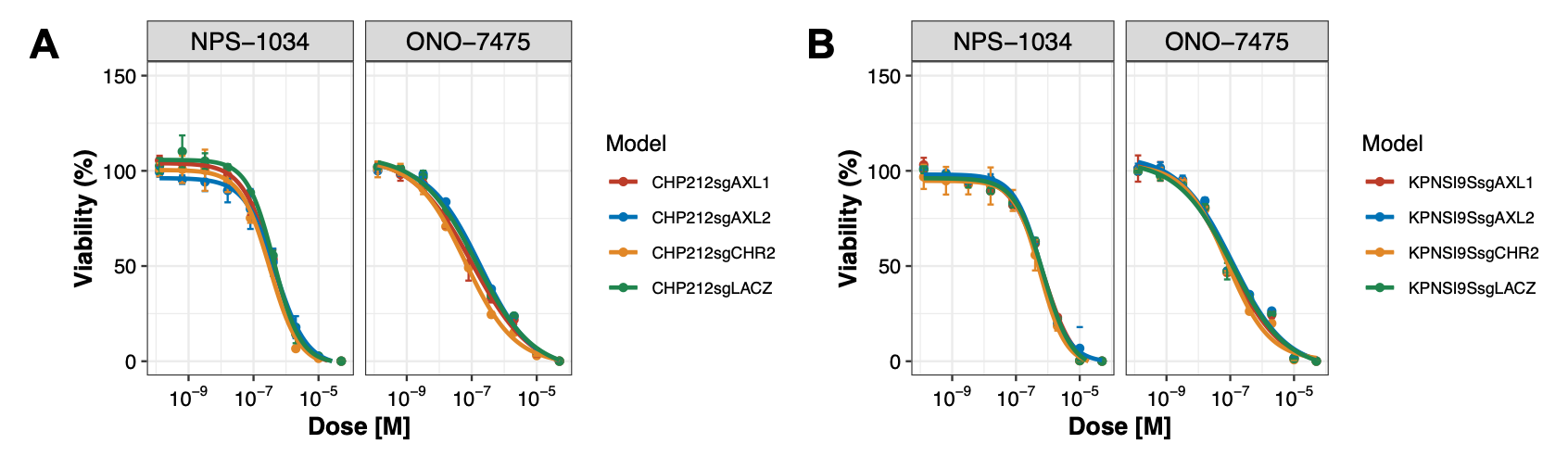 |
| S. Figure 12 Legend: A-B) Cytotoxicity of small molecule AXL inhibitors (NPS-1034 and ONO-7475) in (A) CHP212 and (B) KPNSI9s CRISPR knockout controls (sgLacz, sgCHR2) or AXL knockout (sgAXL1, sgAXL2) models. Viability was measured with CellTiter-Glo2.0 after 3 days of exposure to each inhibitor. Replicates are technical. |

| **Supplemental Figure 13. EGFR- and PDGFR-targeted small molecule inhibitors show modest specificity towards MES neuroblastoma cell line models.** |
| --- |
| 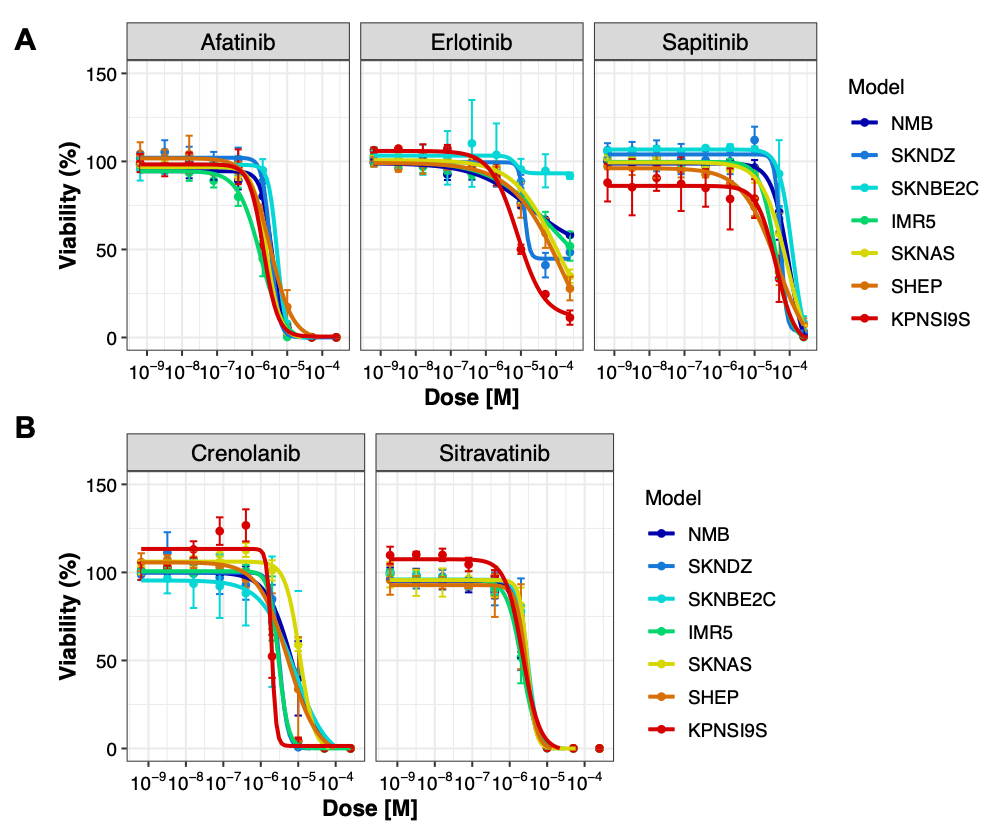 |
| S. Figure 13 Legend: A-B) Cytotoxicity of small molecule inhibitors targeting (A) EGFR and (B) PDGFRA/B across a panel of ADRN and MES neuroblastoma cell lines. Viability was measured with CellTiter-Glo2.0 after 3 days of exposure to each inhibitor after 2-3 independent experiments. |

| **Supplemental Figure 14. ADCT-601 is only active in MES-dominant model, SKNAS.** |
| --- |
| 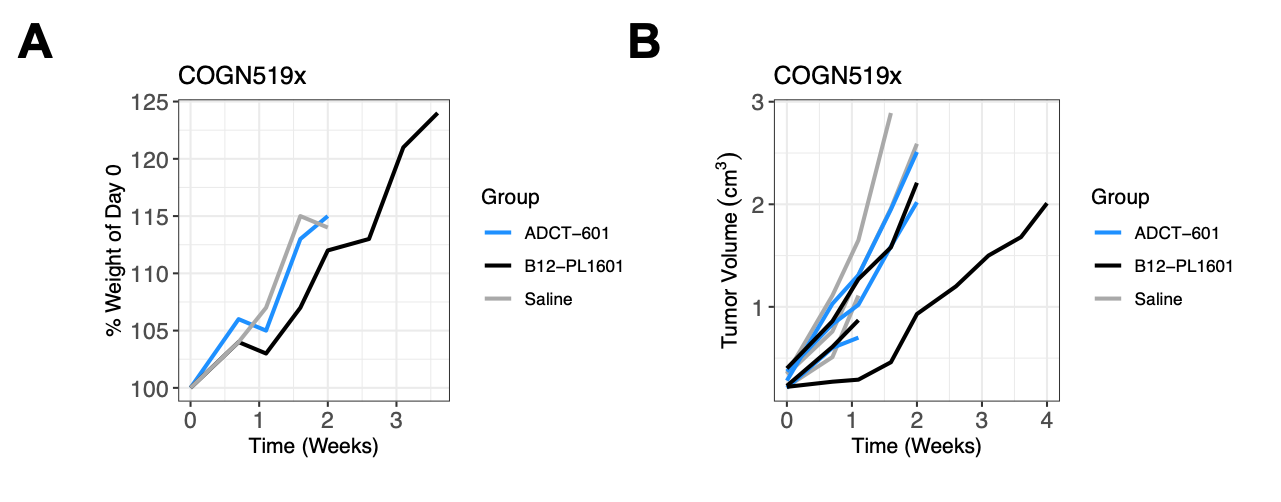 |
| S. Figure 14 Legend: A) Mouse weights after treatment (day 0) as a percent of day 0. B) Tumor volumes after treatment (day 0) of individual mice. Treatments are Saline, Control ADC (B12-PL1601, 1mg/kg dose), or AXL-targeted ADC (ADCT-601, 1mg/kg dose). N=2-3 animals per treatment arm. |

**References for Supplemental Methods**

**1.** Robinson MD, McCarthy DJ, Smyth GK. edgeR: a Bioconductor package for differential expression analysis of digital gene expression data. *Bioinformatics.* 2010; 26(1):139-140.

**2.** McCarthy DJ, Chen Y, Smyth GK. Differential expression analysis of multifactor RNA-Seq experiments with respect to biological variation. *Nucleic Acids Res.* 2012; 40(10):4288-4297.

**3.** Harenza JL, Diamond MA, Adams RN, et al. Transcriptomic profiling of 39 commonly-used neuroblastoma cell lines. *Sci Data.* 2017; 4:170033.

**4.** Rokita JL, Rathi KS, Cardenas MF, et al. Genomic Profiling of Childhood Tumor Patient-Derived Xenograft Models to Enable Rational Clinical Trial Design. *Cell reports.* 2019; 29(6):1675-1689 e1679.

**5.** Mabe NW, Huang M, Dalton GN, et al. Transition to a mesenchymal state in neuroblastoma confers resistance to anti-GD2 antibody via reduced expression of ST8SIA1. *Nat Cancer.* 2022.

**6.** Jansky S, Sharma AK, Korber V, et al. Single-cell transcriptomic analyses provide insights into the developmental origins of neuroblastoma. *Nat Genet.* 2021.

**7.** Dong R, Yang R, Zhan Y, et al. Single-Cell Characterization of Malignant Phenotypes and Developmental Trajectories of Adrenal Neuroblastoma. *Cancer Cell.* 2020; 38(5):716-733 e716.

**8.** Grossmann LD, Chen CH, Uzun Y, et al. Identification and characterization of chemotherapy resistant high-risk neuroblastoma persister cells. *Cancer Discov.* 2024.

**9.** Patel AG, Ashenberg O, Collins NB, et al. A spatial cell atlas of neuroblastoma reveals developmental, epigenetic and spatial axis of tumor heterogeneity. *bioRxiv.* 2024.

**10.** Foroutan M, Bhuva DD, Lyu R, Horan K, Cursons J, Davis MJ. Single sample scoring of molecular phenotypes. *BMC Bioinformatics.* 2018; 19(1):404.

**11.** van Groningen T, Koster J, Valentijn LJ, et al. Neuroblastoma is composed of two super-enhancer-associated differentiation states. *Nat Genet.* 2017; 49(8):1261-1266.

**12.** Law CW, Chen Y, Shi W, Smyth GK. voom: Precision weights unlock linear model analysis tools for RNA-seq read counts. *Genome Biol.* 2014; 15(2):R29.

**13.** Ritchie ME, Phipson B, Wu D, et al. limma powers differential expression analyses for RNA-sequencing and microarray studies. *Nucleic Acids Res.* 2015; 43(7):e47.

**14.** Barry E, Walsh JA, Weinrich SL, et al. Navigating the Regulatory Landscape to Develop Pediatric Oncology Drugs: Expert Opinion Recommendations. *Paediatr Drugs.* 2021; 23(4):381-394.

**15.** Finotello F, Mayer C, Plattner C, et al. Molecular and pharmacological modulators of the tumor immune contexture revealed by deconvolution of RNA-seq data. *Genome Med.* 2019; 11(1):34.

**16.** Racle J, Gfeller D. EPIC: A Tool to Estimate the Proportions of Different Cell Types from Bulk Gene Expression Data. *Methods Mol Biol.* 2020; 2120:233-248.

**17.** Upton K, Modi A, Patel K, et al. Epigenomic profiling of neuroblastoma cell lines. *Sci Data.* 2020; 7(1):116.

**18.** Lalchungnunga H, Hao W, Maris JM, et al. Genome wide DNA methylation analysis identifies novel molecular subgroups and predicts survival in neuroblastoma. *Br J Cancer.* 2022.

**19.** Subramanian A, Tamayo P, Mootha VK, et al. Gene set enrichment analysis: a knowledge-based approach for interpreting genome-wide expression profiles. *Proc Natl Acad Sci U S A.* 2005; 102(43):15545-15550.

**20.** Liberzon A, Birger C, Thorvaldsdottir H, Ghandi M, Mesirov JP, Tamayo P. The Molecular Signatures Database (MSigDB) hallmark gene set collection. *Cell Syst.* 2015; 1(6):417-425.

**21.** Wolpaw AJ, Grossmann LD, Dessau JL, et al. Epigenetic state determines inflammatory sensing in neuroblastoma. *Proc Natl Acad Sci U S A.* 2022; 119(6).

**22.** Sanjana NE, Shalem O, Zhang F. Improved vectors and genome-wide libraries for CRISPR screening. *Nat Methods.* 2014; 11(8):783-784.

**23.** Zammarchi F, Havenith KE, Chivers S, et al. Preclinical Development of ADCT-601, a Novel Pyrrolobenzodiazepine Dimer-based Antibody-drug Conjugate Targeting AXL-expressing Cancers. *Mol Cancer Ther.* 2022; 21(4):582-593.

**24.** Kendsersky NM, Lindsay JM, Kolb EA, et al. The B7-H3-targeting antibody-drug conjugate m276-SL-PBD is potently effective against pediatric cancer preclinical solid tumor models. *Clin Cancer Res.* 2021.
